# pH-Regulated Sequential Ti–Al Incorporation for Enhanced Water Vapor Adsorption and Mechanical Robustness of Silica-Based Desiccants

**DOI:** 10.3390/ma19194030

**Published:** 2026-09-22

**Authors:** Mingyan Wang, Yongcheng Si, Siwei Hu, Yongjun Wu

**Affiliations:** 1 School of Materials Science and Engineering, Institute of Fundamental and Transdisciplinary Research, Zhejiang University, Hangzhou 310027, China; wangmingyanchristy@gmail.com; 2 Hangzhou Dry Air Intelligent Equipment Co., Ltd., Hangzhou 311300, China; siyc126@126.com (Y.S.);

**Keywords:** modified silica gel, sequential doping, pH regulation, mesoporous structure, water vapor adsorption

## Abstract

**Highlights:**

• A pH-mediated sequential Ti–Al incorporation strategy was developed.
• Sequential Ti–Al doping balanced porosity and mechanical robustness.
• pH-mediated sequential doping suppressed Ti precursor aggregation.
• Sequential doping promoted a uniform and interconnected mesoporous network.

**Abstract:**

Silica-based desiccants are widely used in rotary dehumidification systems but are limited by insufficient water adsorption and mechanical durability. Herein, Al–SiO_2_, Ti–SiO_2_, and sequentially co-doped TiAl–SiO_2_ adsorbents were prepared on glass fiber honeycomb substrates to investigate the effects of metal incorporation and pH regulation on pore structure, adsorption performance, and mechanical strength. Al incorporation under mildly acidic conditions (pH 2.5–2.7) promoted the formation of a homogeneous mesoporous network, whereas Ti incorporation under strongly acidic conditions (pH ≈ 1.0) caused rapid precursor hydrolysis, resulting in the aggregation of Ti-containing species, pore blockage, and deteriorated adsorption performance despite improved mechanical strength. A pH-mediated sequential incorporation strategy was therefore developed by introducing Ti while maintaining the reaction pH at approximately 2.0, effectively suppressing uncontrolled hydrolysis and promoting uniform Ti dispersion within the aluminosilicate framework. The resulting TiAl–SiO_2_ exhibited a specific surface area of 460 m^2^ g^−1^, a pore volume of 0.37 cm^3^ g^−1^, a low fractal dimension of 2.60, and an equilibrium water uptake of 23.08%, while maintaining a compressive strength of 1.25 MPa. These findings demonstrate that regulating precursor hydrolysis through pH control provides an effective strategy for tailoring heterometal incorporation, simultaneously optimizing pore structure and mechanical robustness, and highlighting the potential of the prepared materials as candidate desiccants for rotary dehumidification applications.

## 1. Introduction

Moisture removal from air is essential for a wide range of industrial and environmental control applications, including heating, ventilation, and air-conditioning (HVAC) systems, industrial drying, pharmaceutical storage, lithium battery manufacturing, and humidity-controlled cleanrooms. Among various solid desiccants, silica gel (SiO_2_) remains one of the most widely used commercial adsorbents owing to its low cost, relatively low regeneration temperature, excellent chemical stability, and favorable adsorption kinetics under moderate humidity conditions [1,2,3,4]. In particular, silica gel-coated rotary desiccant wheels have been widely adopted in HVAC systems because of their energy-saving potential and operational simplicity [5,6,7]. Despite these advantages, the adsorption performance of conventional silica gel is still constrained by several intrinsic limitations. Its equilibrium water uptake gradually approaches a plateau under medium-to-high relative humidity and remains lower than that of recently developed composite desiccants and metal–organic frameworks (MOFs) [8,9]. Moreover, prolonged exposure to humid regeneration environments may induce gradual hydrothermal aging and pore structure evolution, resulting in a slow decline in long-term adsorption performance. For silica-coated rotary desiccants, inadequate coating integrity and mechanical robustness may further cause coating cracking, particle shedding, and reduced service life during repeated thermal and airflow cycling [10,11]. To overcome these limitations, considerable efforts have been devoted to improving silica-based desiccants through pore structure engineering, surface chemical modification, and compositional regulation. These strategies aim to simultaneously enhance water adsorption capacity, mass-transfer efficiency, hydrothermal stability, and mechanical durability, thereby meeting the increasing performance requirements of next-generation dehumidification systems.

Metal-ion doping has emerged as an effective strategy for regulating the pore structure and surface chemistry of silica adsorbents. Among various dopants, aluminum has attracted significant attention because Al^3+^ can be incorporated into silica frameworks through Si–O–Al bond formation, which increases surface acidity, improves hydrophilicity, and promotes mesoporous development. Consequently, Al-modified silica materials generally exhibit increased specific surface area, enlarged pore volume, and enhanced water adsorption performance. For example, Fang et al. reported that Al-modified silica gels prepared via co-precipitation exhibited improved water adsorption capacity and hydrothermal stability compared with pure silica gel [12]. Similarly, Okada et al. demonstrated that the incorporation of Al species into silica frameworks significantly increased the specific surface area (up to 564 m^2^ g^−1^) and pore volume of aluminosilicate xerogels, providing favorable porous structures for adsorption applications [13]. On the other hand, tetravalent titanium (Ti^4+^) has been explored for its ability to potentially improve the structural rigidity and mechanical stability of silica frameworks. However, compared with Al^3+^, Ti^4+^ possesses substantially faster hydrolysis kinetics, making homogeneous incorporation into the silica network considerably more challenging. The mismatch between the hydrolysis rates of Ti and silicate species frequently results in premature nucleation and aggregation of Ti-containing species, leading to phase separation, pore blockage, reduced surface area, and deterioration of adsorption performance [14,15,16]. Although previous studies have demonstrated the potential of single-metal doping, two major challenges remain unresolved. First, achieving uniform incorporation of heterometal species without inducing precursor aggregation or pore collapse remains difficult. Second, improving adsorption capacity often comes at the expense of mechanical stability, while enhancing structural rigidity frequently reduces accessible porosity. Balancing these competing requirements remains a major bottleneck for practical desiccant applications. In many prior studies, the role of solution acidity (i.e., the pH of the metal-salt precursor solution) and the sequence of dopant addition have not been systematically explored, yet they are critical parameters for controlling ion hydrolysis, condensation, precipitate nucleation, and thus the ultimate pore structure and dopant distribution. For instance, in the co-precipitation of titanium-containing silicates, the precipitation behavior depends strongly on pH, with Ti species precipitating or segregating under acidic conditions if hydrolysis is too rapid [17]. By contrast, a more moderate pH may slow hydrolysis, thus favoring incorporation of Ti into the silica network rather than forming separate TiO_2_ phases. Therefore, regulating the hydrolysis kinetics of heterometal precursors, rather than simply adjusting dopant composition, is expected to provide a more effective route for simultaneously controlling pore architecture, dopant distribution, and the resulting adsorption performance.

To address these challenges, this study proposes a pH-regulated sequential doping strategy for preparing Ti–Al modified silica desiccants. Aluminum sulfate was first introduced to construct a mesoporous aluminosilicate framework under mildly acidic conditions, followed by the slow addition of titanium sulfate while maintaining a buffered pH environment to suppress the rapid hydrolysis of Ti species. This strategy was designed to regulate the introduction of Ti species into the evolving silica–alumina network through controlled pH and sequential addition, thereby promoting their homogeneous distribution and minimizing the formation of Ti-containing aggregates that could restrict pore development and accessibility. The influences of Al doping, Ti doping, and sequential Ti–Al co-doping on morphology, pore structure evolution, fractal characteristics, water vapor adsorption behavior, and mechanical properties were systematically investigated. In this way, the proposed strategy addresses the dual challenge of homogeneous dopant incorporation and pore structure optimization, providing a simple and effective approach for regulating heterometal incorporation through hydrolysis-kinetics control and offering new insights into the design of high-performance silica-based desiccants for advanced rotary dehumidification applications.

## 2. Experimental

### 2.1. Materials

Glass fiber paper (Shaanxi Metop Environmental Protection., Ltd., Xi’an, China) with an areal density of 25 g·m^−2^ was used as the supporting substrate. Sodium silicate aqueous solution (40%, Foshan Zhongfa Water Glass Factory, Foshan, China), and sulfuric acid (95–98%, SCR, Shanghai, China) were used as received without further purification. Al_2_(SO_4_)_3_ (99.5%, Macklin, Shanghai, China) and Ti (SO_4_)_2_ (≥96%, Macklin, China) were used as the precursors.

### 2.2. Preparation of Metal-Modified Silica Adsorbents on Glass Fiber Paper

A fiberglass honeycomb block, fabricated from corrugated glass fiber paper, was directly used as the impregnation substrate. It was first dried in an oven at 100 °C until a constant mass was obtained to remove residual moisture. After drying, the samples were sealed and cooled to room temperature to minimize moisture adsorption from the ambient environment.

The dried fiberglass honeycomb block was then immersed in a 40 wt.% sodium silicate solution for 5 min to ensure complete impregnation of the silicate precursor throughout the fibrous framework. After impregnation, the samples were removed and dried at room temperature for 2 h, allowing uniform deposition of sodium silicate within the substrate. Subsequently, the sodium silicate–impregnated fiberglass honeycomb block was subjected to an acid-induced gelation and metal incorporation process. For Al-modified silica adsorbents, the samples were immersed in a 10 wt.% aluminum sulfate solution with an initial pH of 2.5–2.7 and activated at 45 °C for 1 h. For Ti-modified silica adsorbents, the impregnated samples were immersed in a 10 wt.% titanium sulfate solution with an initial pH of approximately 1.0 and activated under the same conditions. After activation, all samples were thoroughly washed with deionized water until the pH of the washing solution became neutral, ensuring the removal of residual sulfate ions and unreacted species. The washed adsorbents were subsequently dried at 100 °C until a constant mass was achieved. The obtained sample was denoted as Al-SiO_2_ and Ti-SiO_2_.

### 2.3. pH-Mediated Sequential Incorporation Strategy for Al–Ti Co-Modified Silica

The sodium silicate–impregnated fiberglass honeycomb block was first immersed in a 10 wt.% aluminum sulfate solution and activated at 45 °C. During the activation process, the neutralization reaction between sodium silicate and aluminum sulfate led to a gradual increase in the solution pH. To maintain the reaction pH at approximately 2.0, a 10 wt.% titanium sulfate solution was manually added dropwise at an approximately uniform rate using a rubber-bulb dropper. Throughout the addition process, the solution pH was continuously monitored using a calibrated pH meter and maintained within a tolerance range of 1.9–2.1. The addition rate of the titanium sulfate solution was manually adjusted according to the real-time pH reading to avoid abrupt fluctuations in solution acidity. After the required titanium sulfate solution had been added, the reaction was allowed to proceed until no further measurable change in pH was observed. The activation process was maintained at 45 °C for a total of 1 h to ensure sufficient metal–silica interaction and incorporation of Al and Ti species into the adsorbent structure. After activation, the samples were washed with deionized water until neutral pH was reached and subsequently dried at 100 °C to constant weight. The obtained co-modified sample was denoted as TiAl–SiO_2_.

For comparison, a pure silica adsorbent without metal modification was synthesized following the same sodium silicate impregnation and acid-induced gelation procedure, excluding the addition of aluminum sulfate or titanium sulfate. This sample was denoted as SiO_2_ and used as a reference to evaluate the effects of metal incorporation and pH regulation on the structural, adsorption, and mechanical properties of the prepared adsorbents.

The adsorbent loading was determined from the mass difference in the fiberglass honeycomb substrate before and after adsorbent deposition and drying. For all prepared samples, the adsorbent loading accounted for approximately 50–60 wt.% of the total composite mass.

### 2.4. Characterization Methods

The surface area, total pore volume, and average pore diameter of the prepared adsorbents were determined using nitrogen adsorption/desorption curves recorded on an ASAP 2460 apparatus (Micromeritics Instrument Co., Norcross, GA, USA) at −196 °C. Before the analysis, the samples were degassed for 12 h at 110 °C in vacuum. The specific surface area was calculated using the Brunauer–Emmett–Teller (BET) method, while the pore size distribution and average pore diameter were determined using the Barrett–Joyner–Halenda (BJH) method based on the adsorption branch of the nitrogen isotherms, assuming cylindrical pore geometry. The total pore volume was determined from the nitrogen uptake near saturation. Scanning electron microscopy (SEM) images were obtained using a ZEISS GeminiSEM 360 (ZEISS, Oberkochen, Germany) to investigate the morphology and microstructural features of the adsorbents. The mechanical robustness of the prepared adsorbents was evaluated by compressive strength testing using STM-S2.1 (Citema Instrument Co., Ltd., Shenzhen, China). Compressive strength was measured using block-shaped samples with dimensions of 3 cm (length) × 3 cm (width) × 3 cm (height). After the samples reached adsorption saturation, a circular loading plate (1.5 cm in diameter) was placed on the sample surface. Compression was applied perpendicular to the honeycomb channels at a loading rate of 5 mm min^−1^. The maximum load was recorded while the deformation at any point of the corrugated structure beneath the loading plate remained within 1 mm. Three independent specimens were tested for each sample, and the average compressive strength was reported. In a GPR-2 constant temperature and humidity chamber (ESPEC Environmental Equipment Co., Osaka, Japan), the saturated water vapor adsorption performance of the adsorbents was evaluated under controlled environmental conditions at 25 °C and 70% relative humidity (RH). Prior to testing, samples with identical dimensions were dried at 100 °C until a constant mass was achieved and subsequently cooled in a sealed drying vessel.

The dried samples were then placed in the chamber and during the adsorption process, the total mass of each sample was recorded at 1 min intervals. Adsorption was considered to reach saturation when no significant change in mass was observed in consecutive measurements, indicating adsorption equilibrium. Three independent specimens were tested for each sample. The saturated water vapor adsorption capacity (Q_sat_) was calculated according to the following equation:

(1) $$Q_{sat}=\frac{m_{sat}-m_{0}}{m_{0}}\times 100\%$$

where $m_{0}$ is the initial dry mass of the entire adsorbent-coated fiberglass honeycomb composite, including both the fiberglass substrate and the deposited adsorbent, and $m_{sat}$ is the sample mass at adsorption equilibrium.

## 3. Results and Discussion

### 3.1. Textural Properties and Pore Structure Evolution

Figure 1 shows the nitrogen adsorption–desorption isotherms and corresponding pore size distributions of all samples, while the quantitative structural parameters are summarized in Table 1. All materials exhibited typical type-IV adsorption isotherms with distinct hysteresis loops, confirming the formation of mesoporous structures. SiO_2_ showed a moderate adsorption capacity with a relatively narrow hysteresis loop. Its BET surface area, average pore diameter, and pore volume were measured to be 270 m^2^g^−1^, 3.66 nm, and 0.09 cm^3^g^−1^, respectively.

Significant structural variations were observed after metal doping, indicating that both dopant chemistry and reaction pH played decisive roles in regulating the pore evolution of silica-based adsorbents. As shown in Figure 1 and Table 1, the Al-SiO_2_ sample exhibited substantially enhanced nitrogen adsorption uptake over the entire relative pressure range compared with pure SiO_2_, particularly in the medium-to-high relative pressure region (P/P_0_ > 0.4), where multilayer adsorption and capillary condensation in mesopores became dominant, suggesting the formation of a more developed mesoporous structure [18,19]. The BET surface area increased substantially to 333 m^2^g^−1^, while the corresponding pore volume expanded to 0.27 cm^3^g^−1^. In addition, the adsorption-branch BJH pore size distribution (Figure 1b) showed a broad distribution across the mesopore range rather than a well-defined dominant peak. Compared with pure SiO_2_, Al-SiO_2_ exhibited a higher differential pore volume over a broad mesopore range, consistent with its increased BET surface area and total pore volume. This structural improvement was associated with the mildly acidic reaction environment generated by the aluminum sulfate solution (pH ≈ 2.5–2.7). Under this condition, the hydrolysis and condensation rates of silicate and Al^3+^ species remained relatively balanced, thereby favoring the gradual incorporation of Al into the silica framework through the formation of Si–O–Al linkages. The introduction of Al species distorted the original Si–O–Si network and promoted defect-induced mesopore generation, thereby increasing accessible surface area and pore connectivity [20,21]. Moreover, the enlarged pore volume and highly interconnected mesoporous channels were beneficial for reducing vapor diffusion resistance and improving adsorption accessibility. Similar observations have been reported for aluminosilicate xerogels and Al-modified silica adsorbents, where moderate acidity promotes homogeneous framework evolution and mesopore development [22].

In contrast, the Ti–SiO_2_ sample exhibited significantly suppressed nitrogen adsorption capacity and deteriorated pore characteristics. The BET surface area sharply decreased to 130 m^2^ g^−1^, accompanied by a reduced pore volume of only 0.08 cm^3^ g^−1^ (Table 1). The adsorption isotherm displayed substantially weakened nitrogen uptake in the medium-to-high relative pressure region, while the adsorption-branch BJH pore size distribution showed a substantially lower differential pore volume over the mesopore range compared with the other modified samples (Figure 1b), consistent with its reduced total pore volume. This structural deterioration was mainly attributed to the strongly acidic environment of the titanium sulfate precursor solution (pH ≈ 1.0), under which Ti^4+^ species underwent extremely rapid hydrolysis and condensation reactions. The mismatch between the hydrolysis kinetics of Ti and silicate species led to the premature aggregation of Ti-containing species, which partially blocked the pore channels and disrupted the continuity of the silica framework [17]. Although the average pore diameter showed a slight increase, this phenomenon mainly originated from local pore coalescence or framework collapse rather than optimized mesostructure evolution. Consequently, Ti-only modification resulted in inferior porous characteristics and limited adsorption accessibility.

It is worth noting that the sequentially co-doped TiAl–SiO_2_ sample exhibited the most optimized pore structure among all samples. The BET surface area further increased to 460 m^2^ g^−1^, while the pore volume reached 0.37 cm^3^ g^−1^ with a BJH adsorption average pore diameter of approximately 3.92 nm (Table 1). Compared with the single-metal modified samples, the adsorption isotherm of TiAl–SiO_2_ displayed significantly enhanced nitrogen uptake across the entire pressure range together with a pronounced hysteresis loop (Figure 1a), indicating the formation of a highly developed and interconnected mesoporous architecture. The adsorption-branch BJH pore size distribution further showed a broad mesopore distribution with TiAl–SiO_2_ exhibiting the highest differential pore volume over much of the investigated mesopore range (Figure 1b). This behavior is consistent with its substantially increased BET surface area and total pore volume.

The superior porous properties of TiAl–SiO_2_ were primarily attributed to the pH-regulated sequential incorporation strategy. In this process, Al species were first introduced to construct a relatively stable aluminosilicate framework under mildly acidic conditions. Subsequently, titanium sulfate was slowly added while maintaining the reaction pH at approximately 2.0. This buffered pH environment effectively reduced the instantaneous hydrolysis driving force of Ti^4+^ species and suppressed uncontrolled TiO_2_ nucleation. As a result, Ti species were more uniformly distributed within the pre-formed silica–alumina network, with reduced formation of Ti-containing aggregates. More importantly, the sequential doping strategy spatially and temporally separated the competitive hydrolysis and condensation pathways of heterometallic precursors, thereby avoiding local structural collapse and preserving pore continuity. The synergistic combination of Al-induced mesopore generation and Ti-stabilized framework reinforcement ultimately produced a hierarchically interconnected mesoporous structure with substantially improved pore accessibility and structural stability. These results collectively demonstrate that pH strongly influences the hydrolysis and condensation behavior of heterometal precursors, thereby governing framework evolution and pore structure development in metal-modified silica systems. Excessively strong acidity accelerates Ti precursor aggregation and causes pore blockage, whereas moderately acidic conditions favor homogeneous framework incorporation and mesopore development. Crucially, the pH-mediated sequential doping strategy successfully balances framework incorporation and phase segregation, enabling simultaneous improvements in pore structure uniformity and adsorption accessibility.

To quantitatively evaluate the pore surface heterogeneity and structural complexity of the prepared adsorbents, the fractal dimension (D) was determined from nitrogen adsorption isotherms using the Frenkel–Halsey–Hill (FHH) model. The FHH theory describes multilayer adsorption on heterogeneous surfaces and has been widely employed to characterize the fractal nature of porous materials [23,24]. According to the FHH model, the relationship between the adsorbed nitrogen volume (V) and the relative pressure (P/P_0_) is expressed as:

$$lnV=C+kln\left[ln(\frac{P_{0}}{P})\right]$$

where C is a constant and k is the slope obtained from linear fitting of the FHH plot.

The surface fractal dimension (D) is then calculated from the slope k using the following relationship:

D=k+3

For surface fractal systems, the fractal dimension typically lies in the range of 2 < D < 3. A value approaching 3 indicates a highly irregular and rough surface with strong heterogeneity, while a value closer to 2 corresponds to a smoother and more uniform surface morphology [25].

Figure 2 presents the FHH fractal fitting plots of all samples obtained from the nitrogen adsorption isotherms and the calculated fractal dimensions are summarized in Table 2. Pure SiO_2_ exhibited a relatively high fractal dimension of 2.77, indicating significant surface roughness and pore structure heterogeneity. This result is consistent with its relatively low pore volume (0.09 cm^3^ g^−1^) and moderate specific surface area (270 m^2^ g^−1^) (Table 1), suggesting that the pore network contained a considerable number of irregular surface domains and poorly connected adsorption sites.

After Al incorporation, the fractal dimension decreased to 2.65 (Table 2), indicating a substantial improvement in pore surface uniformity. This reduction was closely associated with the mildly acidic synthesis environment provided by the aluminum sulfate solution (pH ≈ 2.5–2.7). Under these conditions, the hydrolysis and condensation behavior of Al^3+^ and silicate species may favor more homogeneous interactions between Al-containing species and the evolving silica network, thereby contributing to more uniform network development. Consequently, a more ordered mesoporous network was generated, accompanied by a significant increase in BET surface area to 333 m^2^ g^−1^ and pore volume to 0.27 cm^3^ g^−1^ (Table 1). The simultaneous decrease in fractal dimension and increase in textural properties suggest that Al incorporation not only expanded the accessible pore network but also reduced structural irregularities within the silica framework.

On the other hand, the Ti–SiO_2_ sample exhibited a fractal dimension of 2.78 (Table 2), which was comparable to that of SiO_2_ despite its substantially reduced surface area and pore volume. This observation indicates that Ti incorporation under strongly acidic conditions (pH ≈ 1.0) did not improve pore surface uniformity. Instead, rapid hydrolysis of Ti^4+^ species promoted the formation of Ti-containing aggregates and structural defects. These aggregates partially blocked pore channels and disrupted framework continuity, resulting in a porous structure characterized by both low accessibility and high surface heterogeneity. The persistence of a high fractal dimension therefore provides quantitative evidence that uncontrolled Ti hydrolysis increased structural disorder rather than improving mesopore organization.

Significantly, the TiAl–SiO_2_ sample exhibited the lowest fractal dimension among all materials (D = 2.60) (Table 2), indicating the highest degree of pore surface regularity and structural homogeneity. This result strongly supports the effectiveness of the pH-mediated sequential incorporation strategy. By introducing Al species first and subsequently adding titanium sulfate while maintaining the reaction pH at approximately 2.0, the hydrolysis behavior of Ti^4+^ species was effectively moderated. The buffered pH environment suppressed rapid aggregation of Ti-containing species and enabled Ti species to be incorporated into the pre-formed aluminosilicate framework in a highly dispersed state.

The evolution of fractal dimension showed excellent agreement with the trends observed in BET surface area, pore volume, and pore size distribution. Specifically, lower fractal dimensions corresponded to higher surface areas, larger pore volumes, and more developed accessible mesoporosity. Such characteristics are highly favorable for water vapor diffusion and adsorption because they reduce tortuosity and facilitate mass transfer within the pore network. Conversely, higher fractal dimensions were associated with greater pore-surface heterogeneity, which could reduce the accessibility of adsorption sites. Therefore, fractal analysis provides additional quantitative evidence that pH-regulated sequential doping not only tailors pore size and pore volume but also fundamentally improves pore surface uniformity and structural order.

### 3.2. Morphology of Adsorbent Coatings on Glass Fiber Substrates

The surface morphologies of the glass fiber substrate and the corresponding metal-modified silica composites were further characterized by SEM, and the results are presented in Figure 3. The pristine glass fiber substrate consists of randomly oriented filamentous filaments with irregular diameters, forming abundant inter-fiber voids and loosely packed channels (Figure 3a). Such a structure provides sufficient space for the deposition of active adsorbent phases while preserving the low-flow-resistance characteristics required for rotary dehumidification applications.

After sodium silicate impregnation and acid-induced gel formation, noticeable changes in surface morphology were observed. For SiO_2_, Al-SiO_2_, and TiAl-SiO_2_ samples, silica-based deposits were uniformly distributed over the fiber surfaces and within the inter-fiber regions. A continuous coating layer was formed, and the original fibrous texture became progressively smoother after loading. Among these samples, Al–SiO_2_ (Figure 3c) and TiAl–SiO_2_ (Figure 3e) exhibited the most homogeneous coating morphology, with no obvious large-particle accumulation observed on the fiber surfaces. The deposited phase appeared well anchored to the substrate and was evenly dispersed throughout the fibrous framework. The relatively uniform coating observed in the SEM images suggests that the mildly acidic environment of the aluminum sulfate solution (pH 2.5–2.7) enabled a controlled gelation process, allowing the silica species to deposit gradually on the fiber surface while maintaining structural continuity. The TiAl–SiO_2_ sample exhibited a similarly uniform morphology (Figure 3e), despite the introduction of titanium species. In contrast to the Ti-only system, no obvious particulate clusters were observed, and the coating layer remained continuous across the fiber surface. This observation suggests that the sequential incorporation route effectively moderated the deposition behavior of Ti-containing species.

However, the Ti-doped sample exhibited significantly different morphological characteristics. The fiber surfaces became noticeably rougher, and numerous micron-scale aggregates were observed both on the fiber surface and within the inter-fiber voids. Instead of forming a continuous coating layer, the deposited phase exhibited an irregular morphology (Figure 3d). This heterogeneous distribution is attributed to the strong acidity of the titanium sulfate solution (pH ≈ 1.0), which accelerates the hydrolysis and condensation of Ti species. Such conditions may promote the aggregation of Ti-containing species, resulting in a less homogeneous structure and reduced pore accessibility. The morphological differences observed by SEM correlate well with the evolution of the pore structure characterized by BET and fractal analyses. Samples exhibiting continuous and homogeneous coatings (Al–SiO_2_ and TiAl–SiO_2_) also showed larger surface areas, higher pore volumes, and lower fractal dimensions, whereas the aggregated morphology of Ti–SiO_2_ corresponded to reduced surface area and increased structural heterogeneity. These observations indicate that the acidity of the reaction environment influences not only the hydrolysis behavior of metal precursors but also the deposition mode of the adsorbent phase on the fibrous substrate, ultimately affecting the development of the porous architecture.

### 3.3. Mechanical Robustness

The compressive strengths of the prepared samples are summarized in Table 1. Pure SiO_2_ exhibited a compressive strength of 1.17 MPa, reflecting the relatively fragile nature of conventional porous silica frameworks. Owing to the highly porous structure generated during the acid-induced gelation process, the silica network contained numerous voids and weak interparticle connections, which limited its resistance to external compression [26]. After Al incorporation, the compressive strength decreased to 0.97 MPa. This reduction was consistent with the pore structure evolution discussed previously. The Al–SiO_2_ sample exhibited a significantly larger surface area and pore volume than SiO_2_, indicating the formation of a more open and highly developed mesoporous network. Although such structural features are favorable for water vapor adsorption and diffusion, the increased porosity inevitably reduced the density of the load-bearing framework and weakened its resistance to compressive deformation. Ti–SiO_2_ exhibited the highest compressive strength among all samples, reaching 1.31 MPa. The improved mechanical performance may be associated with the structural changes induced by the introduction of Ti-containing species, resulting in enhanced structural rigidity. Moreover, the lower pore volume and reduced mesoporosity of Ti–SiO_2_ resulted in a relatively denser structure, which further contributed to its enhanced resistance to compression. However, SEM observations revealed the presence of localized particle agglomerates and non-uniform coating regions (Figure 3d). Such morphological heterogeneity suggests that the improvement in strength was achieved at the expense of pore structure development. Remarkably, the TiAl–SiO_2_ sample exhibited a compressive strength of 1.25 MPa, which remained substantially higher than that of Al–SiO_2_ while only slightly lower than that of Ti–SiO_2_. Considering that TiAl–SiO_2_ simultaneously possessed the highest BET surface area (460 m^2^ g^−1^) and pore volume (0.37 cm^3^ g^−1^) (Table 1), this result indicates that the introduction of Ti species contributed to framework reinforcement without severely compromising the development of the porous structure. This behavior was closely related to the pH-regulated sequential incorporation strategy employed during synthesis. Al incorporation primarily promotes pore development but reduces framework compactness, whereas Ti incorporation enhances rigidity but suppresses mesopore formation under strongly acidic conditions. By regulating precursor hydrolysis through pH-controlled sequential doping, the Ti–Al co-modified sample achieves a balanced optimization of mechanical strength and porosity, demonstrating that precise control over metal incorporation and dispersion is critical for simultaneously enhancing structural robustness and maintaining high surface area. Such characteristics are particularly desirable for rotary dehumidification applications, where adsorbents are required to withstand long-term cyclic operation while maintaining efficient mass transfer performance.

### 3.4. Water Vapor Adsorption Performance

Figure 4 shows the static water vapor adsorption curves of the prepared adsorbents, and the corresponding equilibrium adsorption capacities are summarized in Table 1. All samples exhibited a rapid increase in adsorption capacity during the initial stage, particularly within the first 200 min, corresponding to the critical fast adsorption period in rotary dehumidification processes. Subsequently, the adsorption rate gradually decreased and approached equilibrium after approximately 300 min, indicating that water transport within the pore network progressively reached a diffusion–adsorption equilibrium state. Such behavior is characteristic of mesoporous silica-based desiccants, where adsorption initially occurs on external active sites and is subsequently governed by pore diffusion, multilayer adsorption, and capillary condensation within the mesoporous structure [27].

The pure SiO_2_ sample reached an equilibrium adsorption capacity of 15.73% (Table 1), with a relatively moderate adsorption rate throughout the process. This behavior can be attributed to its limited BET surface area (270 m^2^ g^−1^) and small pore volume (0.09 cm^3^ g^−1^) (Table 1), which restricted the number of accessible adsorption sites and limited water vapor transport into the interior pore structure, thereby constraining the overall adsorption capacity.

The Al–SiO_2_ sample exhibited a steeper adsorption curve during the initial stage and reached an equilibrium adsorption capacity of 15.62% (Table 1). Although the equilibrium uptake was comparable to that of pure SiO_2_, the adsorption kinetics were accelerated during the initial stage of adsorption. This behavior corresponded well with the development of a more interconnected mesoporous structure observed from the BET analysis. The increase in surface area from 270 to 333 m^2^ g^−1^ and the expansion of pore volume from 0.09 to 0.27 cm^3^ g^−1^ (Table 1) provided additional adsorption sites and shortened the diffusion pathway for water molecules. The formation of mesopores centered within the 3–5 nm (Figure 1b) range is particularly favorable for water vapor transport because it reduces diffusion resistance while maintaining sufficient capillary condensation effects.

By comparison, the Ti–SiO_2_ sample exhibited the lowest adsorption performance among all materials. The equilibrium adsorption capacity decreased to 5.54% (Table 1), and the adsorption curve remained significantly flatter throughout the entire adsorption period (Figure 4). This behavior was consistent with the substantial reduction in BET surface area (130 m^2^ g^−1^) and pore volume (0.08 cm^3^ g^−1^) (Table 1). The diminished adsorption capacity can be attributed to the loss of accessible pore space and reduced connectivity within the porous network. SEM observations further showed the presence of aggregated deposits on the fiber surface, which likely restricted water vapor transport and reduced the availability of effective adsorption sites. The strongly acidic precursor environment (pH ≈ 1.0) accelerated the hydrolysis of titanium species and altered the evolution of the silica framework, resulting in a less developed mesoporous structure compared with the Al-modified sample. Although titanium-containing species may introduce additional hydroxyl functionalities, the loss of accessible pore volume and the increase in diffusion resistance became the dominant factors controlling adsorption performance in this sample.

Among all materials, TiAl–SiO_2_ showed the highest adsorption performance. The equilibrium adsorption capacity reached 23.08% (Table 1), representing an increase of approximately 47% relative to SiO_2_. The adsorption curve displayed the steepest initial slope (Figure 4), indicating rapid water uptake during the early adsorption stage. This superior adsorption performance corresponded closely to its optimized pore characteristics, including the highest BET surface area (460 m^2^ g^−1^), the largest pore volume (0.37 cm^3^ g^−1^), and a BJH adsorption average pore diameter of approximately 3.92 nm (Table 1). More importantly, the variation in fractal dimension was associated with the water vapor adsorption behavior of the adsorbents. As shown in Table 1 and Table 2 and Figure 2, the decrease in fractal dimension from SiO_2_ to Al–SiO_2_ and TiAl–SiO_2_ was accompanied by improved pore accessibility and water adsorption performance. In particular, TiAl–SiO_2_ exhibited the lowest fractal dimension of 2.60 together with the highest equilibrium water uptake of 23.08%. A lower surface fractal dimension generally reflects reduced surface heterogeneity and a more uniform pore interface [28], which, together with the increased pore volume and interconnected mesoporous structure, can facilitate water-vapor transport and improve the accessibility of adsorption sites. The combination of abundant adsorption sites and highly interconnected mesoporous channels facilitated rapid diffusion of water molecules from the external surface to the internal pore structure, thereby improving both adsorption kinetics and equilibrium uptake.

To further elucidate the relationship between water vapor adsorption performance and the pore structural characteristics of the adsorbents, the saturated adsorptive ratio was compared with the pore volume, BET specific surface area, average pore diameter, and compressive strength, as shown in Figure 5. SiO_2_ exhibited a relatively high water uptake of 15.73%; however, its pore structure remained underdeveloped, as reflected by its limited BET specific surface area (270 m^2^ g^−1^) and pore volume (0.09 cm^3^ g^−1^). Metal incorporation substantially altered this structure–property relationship, although the effect strongly depended on the incorporated metal species and synthesis conditions. In particular, In particular, Al–SiO_2_ and TiAl–SiO_2_ exhibited more developed porous structures than SiO_2_; however, only TiAl–SiO_2_ showed a substantial increase in equilibrium water uptake, indicating that water adsorption was not governed by a single textural parameter.

The adsorption capacity generally followed the variations in pore volume and BET specific surface area (Figure 5a,b). TiAl–SiO_2_, which possessed the largest pore volume (0.37 cm^3^ g^−1^) and BET specific surface area (460 m^2^ g^−1^), exhibited the highest water uptake (23.08%). By contrast, Ti–SiO_2_ showed the lowest accessible surface area and pore volume and correspondingly the lowest adsorption capacity (5.54%). Notably, although Ti–SiO_2_ possessed the largest average pore diameter, its water uptake remained substantially lower than that of SiO_2_ (Figure 5c), demonstrating that pore enlargement alone did not necessarily improve adsorption when the accessible surface area and pore volume were simultaneously reduced. The comparison with SiO_2_ therefore further indicates that the beneficial effect of metal incorporation originates from the development of accessible porosity rather than from pore enlargement itself. A similarly direct relationship was not observed between compressive strength and water uptake (Figure 5d). Ti–SiO_2_ exhibited the highest compressive strength but the lowest adsorption capacity, whereas Al–SiO_2_ showed improved adsorption despite its lower compressive strength. In comparison, TiAl–SiO_2_ maintained relatively high compressive strength while achieving the highest adsorption capacity. These results suggest that accessible surface area and pore volume are more closely associated with water uptake than average pore diameter, whereas mechanical strength represents an important engineering requirement rather than a direct determinant of adsorption capacity. Accordingly, the sequential Ti–Al modification achieved a favorable combination of developed porosity, enhanced water adsorption, and mechanical robustness.

Overall, the adsorption behavior followed the same trend as the evolution of pore structure parameters:

TiAl-SiO_2_ > SiO_2_ ≈ Al-SiO_2_ > Ti-SiO_2_

indicating that water vapor adsorption was governed by the combined effects of pore structure development and pore surface uniformity generated under different pH-controlled synthesis conditions. Strongly acidic conditions induce uncontrolled Ti hydrolysis and pore blockage, while mildly acidic environments promote uniform Al incorporation and mesopore development. In contrast, the buffered pH environment employed in the sequential co-doping strategy effectively suppresses heterometal precursor aggregation and enables cooperative framework construction. Al species were first incorporated into the silica framework under mildly acidic conditions, followed by the gradual introduction of titanium sulfate while maintaining the reaction pH near 2.0. Compared with the Ti-only system, the moderated acidity altered the hydrolysis behavior of titanium species and enabled their incorporation into the evolving aluminosilicate network without significantly disturbing pore development. Consequently, the resulting material retained the mesoporous characteristics associated with Al incorporation while benefiting from the presence of Ti species within the framework.

## 4. Conclusions

This study systematically investigated the effects of Al doping, Ti doping, and pH-mediated sequential Ti–Al incorporation on the pore structure evolution, mechanical robustness, and water vapor adsorption performance of silica-based adsorbents. The results suggest that differences in reaction pH may influence the hydrolysis behavior of metal precursors, thereby contributing to the observed variations in determining metal dispersion, pore architecture, and ultimately the adsorption performance of the prepared materials. Mildly acidic conditions favor the homogeneous incorporation of Al species and the development of interconnected mesopores, whereas the rapid hydrolysis of Ti precursors under strongly acidic conditions may promote the aggregation and non-uniform deposition of Ti-containing species, thereby restricting pore development and accessibility, resulting in deteriorated porous characteristics despite improved mechanical strength. By introducing Ti species through a pH-mediated sequential incorporation strategy, uncontrolled Ti hydrolysis is effectively suppressed, enabling uniform Ti dispersion within the pre-formed aluminosilicate framework. Consequently, the TiAl–SiO_2_ adsorbent exhibits the highest BET surface area (460 m^2^ g^−1^), pore volume (0.37 cm^3^ g^−1^), equilibrium water adsorption capacity (23.08%), and a relatively high compressive strength (1.25 MPa), together with the lowest fractal dimension (2.6), indicating a highly accessible and structurally homogeneous mesoporous network. This work establishes pH-regulated precursor hydrolysis as a practical approach for controlling heterometal incorporation and pore structure evolution, providing a feasible strategy for developing silica-based desiccants with simultaneously enhanced adsorption capacity and mechanical durability.

## Figures and Tables

**Figure 1:**
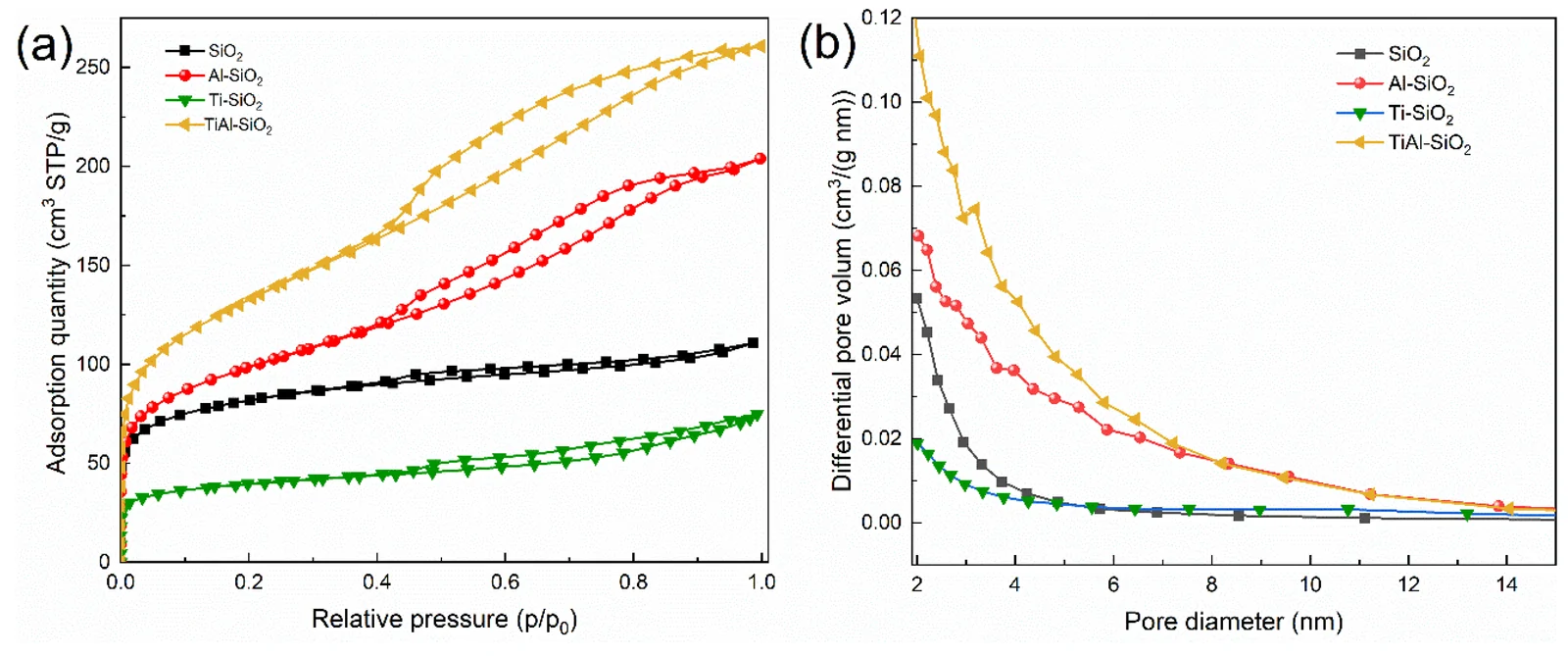
(**a**) Nitrogen adsorption–desorption isotherm and (**b**) pore size distribution of SiO_2_ and the modified silica gels.

**Figure 2:**
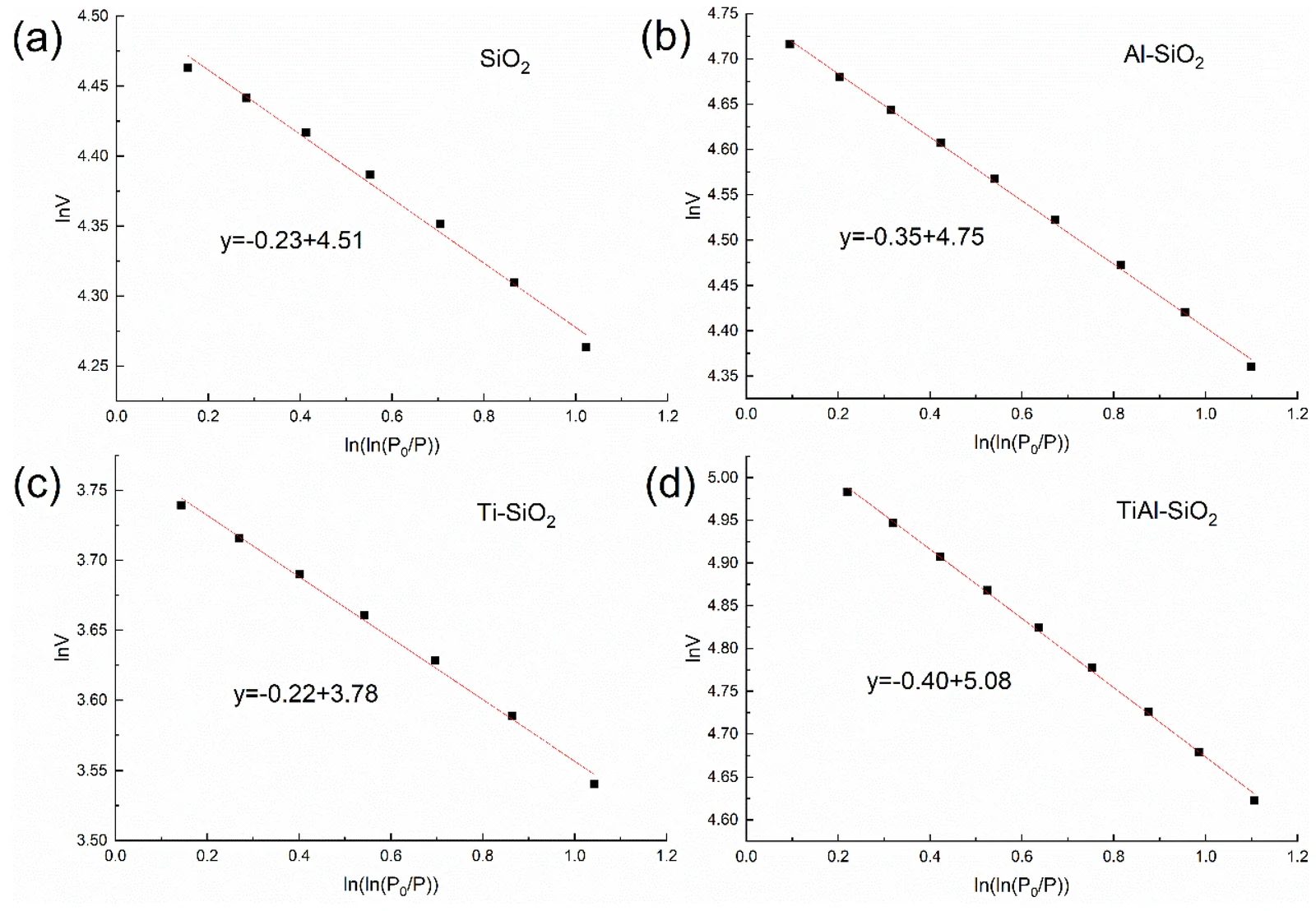
The measure of the fractal dimensions for (**a**) SiO_2_, (**b**) Al-SiO_2_, (**c**) Ti-SiO_2_, and (**d**) TiAl-SiO_2_.

**Figure 3:**
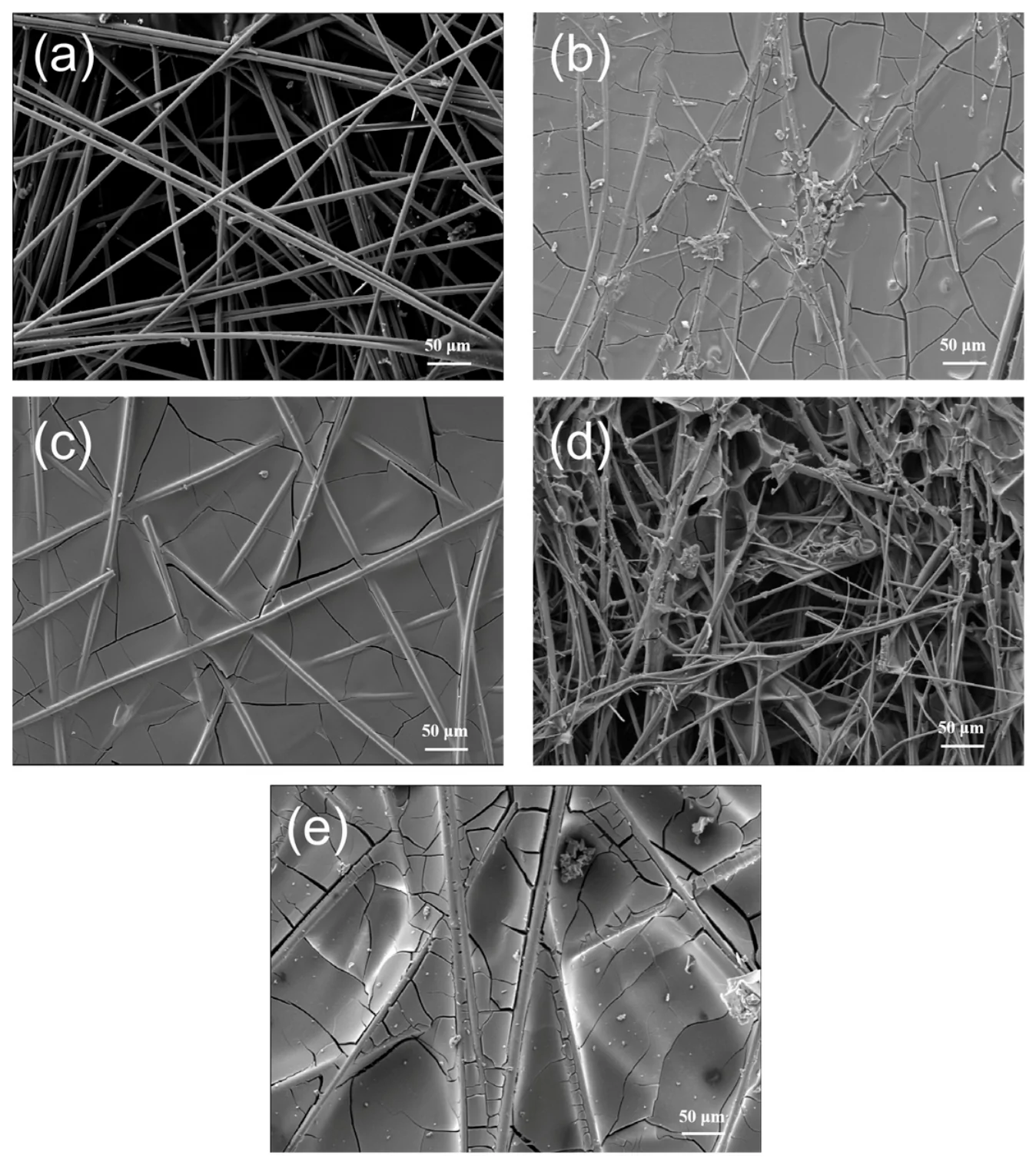
SEM images of (**a**) pristine glass fiber, (**b**) SiO_2_, (**c**) Al-SiO_2_, (**d**) Ti-SiO_2_, and (**e**) TiAl-SiO_2_.

**Figure 4:**
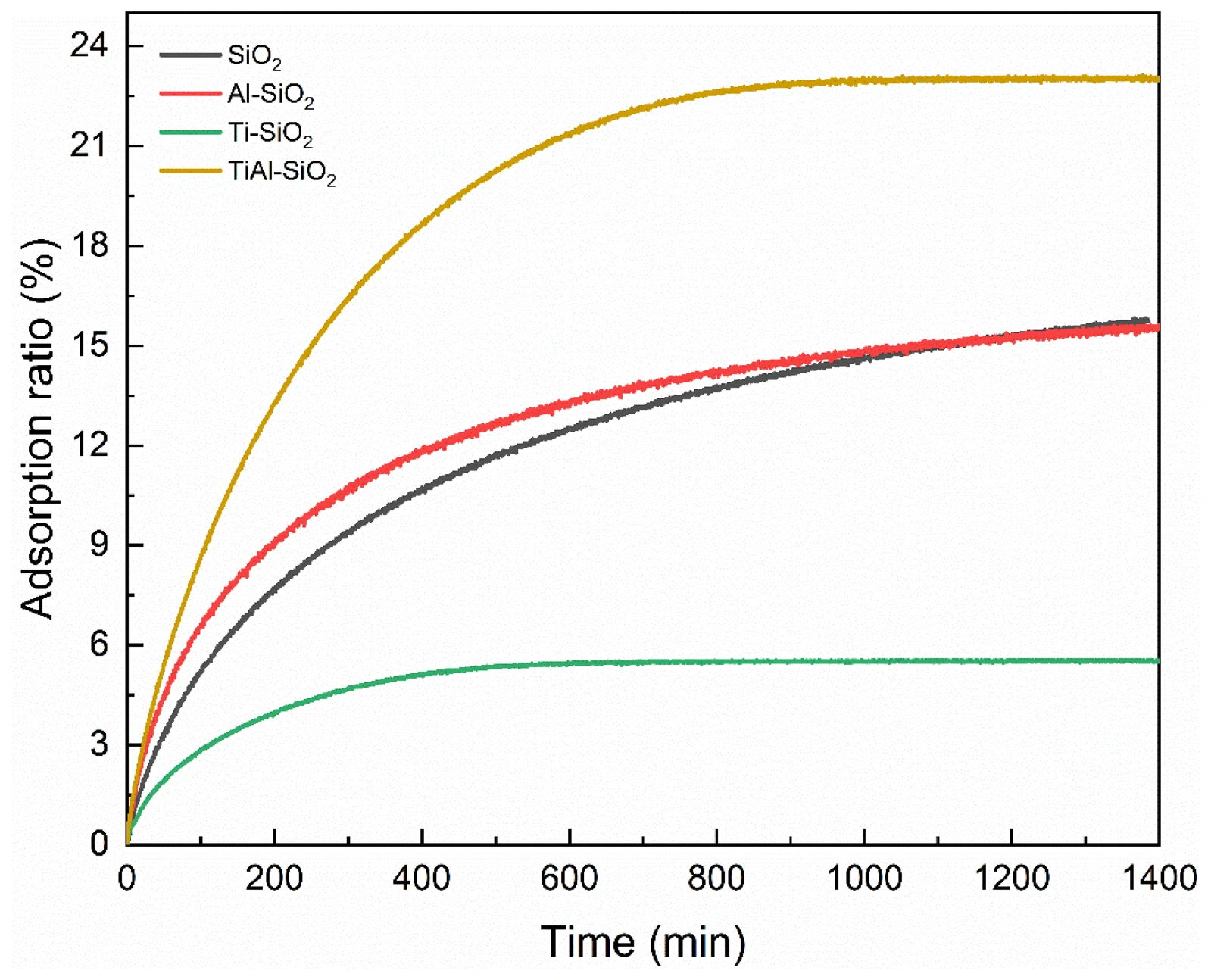
Static adsorption curves of SiO_2_ and the modified silica gels.

**Figure 5:**
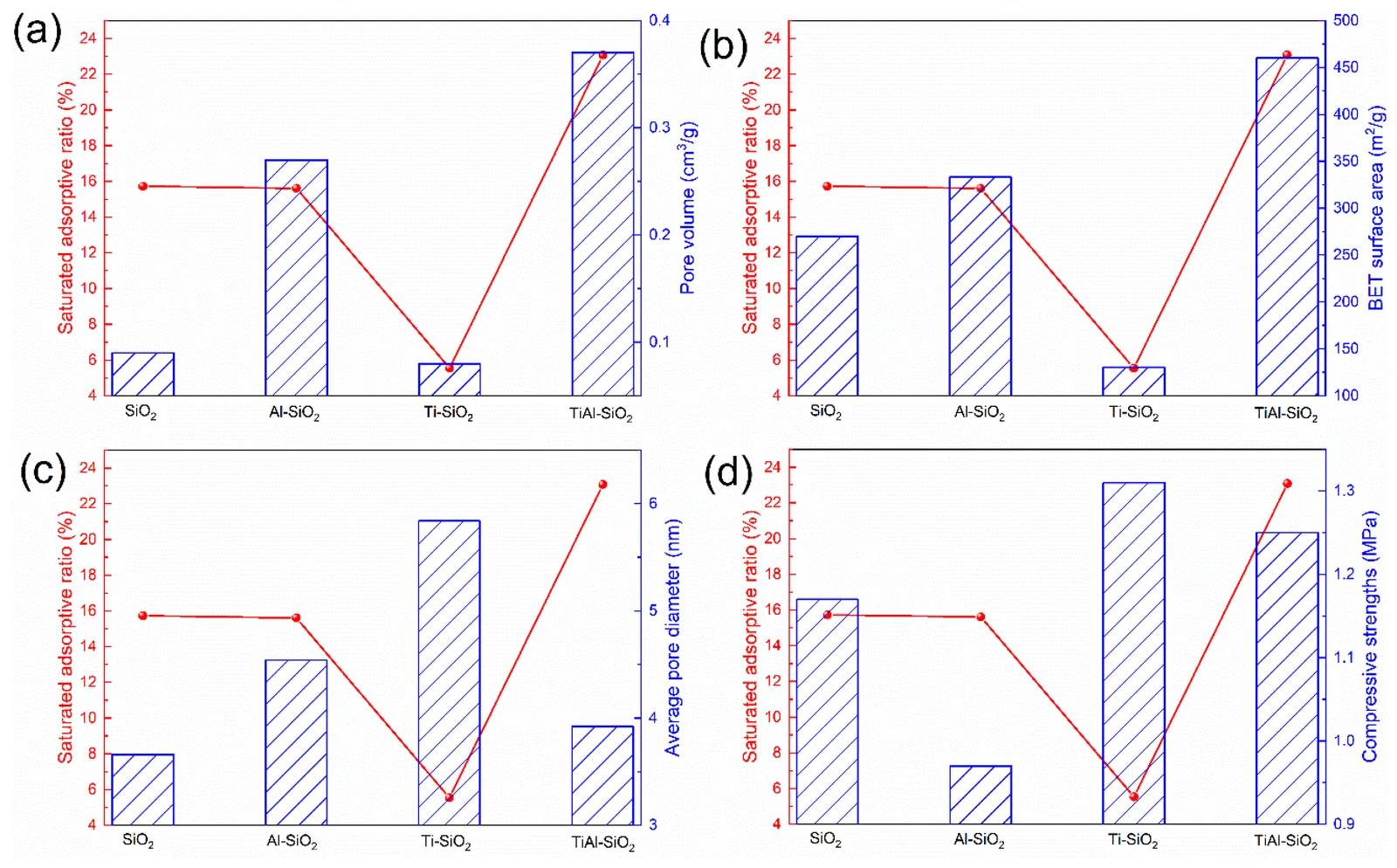
Relationships between the maximum water vapor adsorption capacity at 1400 min and (**a**) pore volume, (**b**) BET specific surface area, (**c**) average pore diameter, and (**d**) compressive strength of SiO_2_ and the modified silica gels.

**Table 1:** Parameters of different composite adsorbents.

Samples	BET Surface Area (m^2^/g)	Average Pore Diameter (nm)	Pore Volume (cm^3^/g)	Compressive Strengths (MPa)	Saturated Adsorptive Ratio (%)
SiO_2_	270	3.66	0.09	1.17	15.73
Al-SiO_2_	333	4.54	0.27	0.97	15.62
Ti-SiO_2_	130	5.84	0.08	1.31	5.54
TiAl-SiO_2_	460	3.92	0.37	1.25	23.08

**Table 2:** The fractal dimension of different composite adsorbents.

Samples	Fractal Dimension
SiO_2_	2.77
Al-SiO_2_	2.65
Ti-SiO_2_	2.78
TiAl-SiO_2_	2.60

## Data Availability

The original contributions presented in this study are included in the article. Further inquiries can be directed to the corresponding author.
